# From *Plasmodium vivax* outbreak to elimination: lessons learnt from a retrospective analysis of data from Guantang

**DOI:** 10.1186/s12936-020-03501-4

**Published:** 2020-11-23

**Authors:** Guo-Jing Yang, Ying Liu, Le-Yuan Shang, Hong-Wei Zhang, Xiao-Nong Zhou, Melissa A. Penny, Thomas A. Smith

**Affiliations:** 1 Swiss Tropical and Public Health Institute, Socinstrasse 57, 4002 Basel, Switzerland; 2 University of Basel, Basel, Switzerland; 3 Laboratory of Tropical Environment and Health, Hainan Medical University, Haikou, Hainan People’s Republic of China; 4 Henan Center for Disease Control and Prevention, Zhengzhou, Henan People’s Republic of China; 5 Chinese Center for Disease Control and Prevention, National Institute of Parasitic Diseases, Shanghai, People’s Republic of China; 6 Key Laboratory of Parasite and Vector Biology, WHO Collaborating Center for Tropical Diseases, Ministry of Health, Shanghai, People’s Republic of China

**Keywords:** *Plasmodium vivax*, Central China, Pattern, Elimination

## Abstract

**Background:**

Malaria was once a serious public health problem in China, with *Plasmodium vivax* the major species responsible for more than 90% of local transmission. Following significant integrated malaria control and elimination programmes, malaria burden declined, and since 2017 China has not recorded any indigenous case. To understand the historical malaria transmission patterns and epidemic characteristics in China and insights useful to guide *P. vivax* malaria control and elimination elsewhere, a retrospective study was carried out.

**Methods:**

Historical data from a pilot study conducted in Guantang, Luyi in central China from 1971–1995, were digitized. The data included monthly numbers of reported cases, febrile cases, parasite carriage rates, the neonatal infection rate, and entomological data regarding *Anopheles sinensis*.

**Results:**

Following 25 years of continuous integrated malaria control activities, malaria incidence in Guantang decreased from 4,333 cases per 10,000 in 1970 before integrated implementation to 0.23 cases per 10,000 in 1991, and no cases in 1992–1995. Some fluctuations in incidence were observed between 1977 and 1981. During the period parasite rates, antibody levels and the neonatal infection rate also decreased. The pattern of seasonality confirmed that *P. vivax* in Henan Province was primarily of the long incubation type (temperate) during non-transmission period. The findings retrospectively provide a scientific basis for the implementation of mass campaigns of liver stage hypnozoite clearance. Entomological studies indicated that *An. sinensis* was the only vector, and it preferred bovine to human hosts, predominantly biting and resting outdoors*.* Mosquito densities declined between 1971 and 1984.

**Conclusion:**

The integrated malaria control approach in Guantang effectively controlled malaria and achieved elimination. Analysis of the effectiveness of the programme can provide guidance to other regions or countries with similar ecological settings aiming to move from malaria control to elimination. There is a potential challenge in the maintenance of non-transmission status owing to imported cases and the long dormancy of liver stage hypnozoites.

## Background

**Fig. 1:**
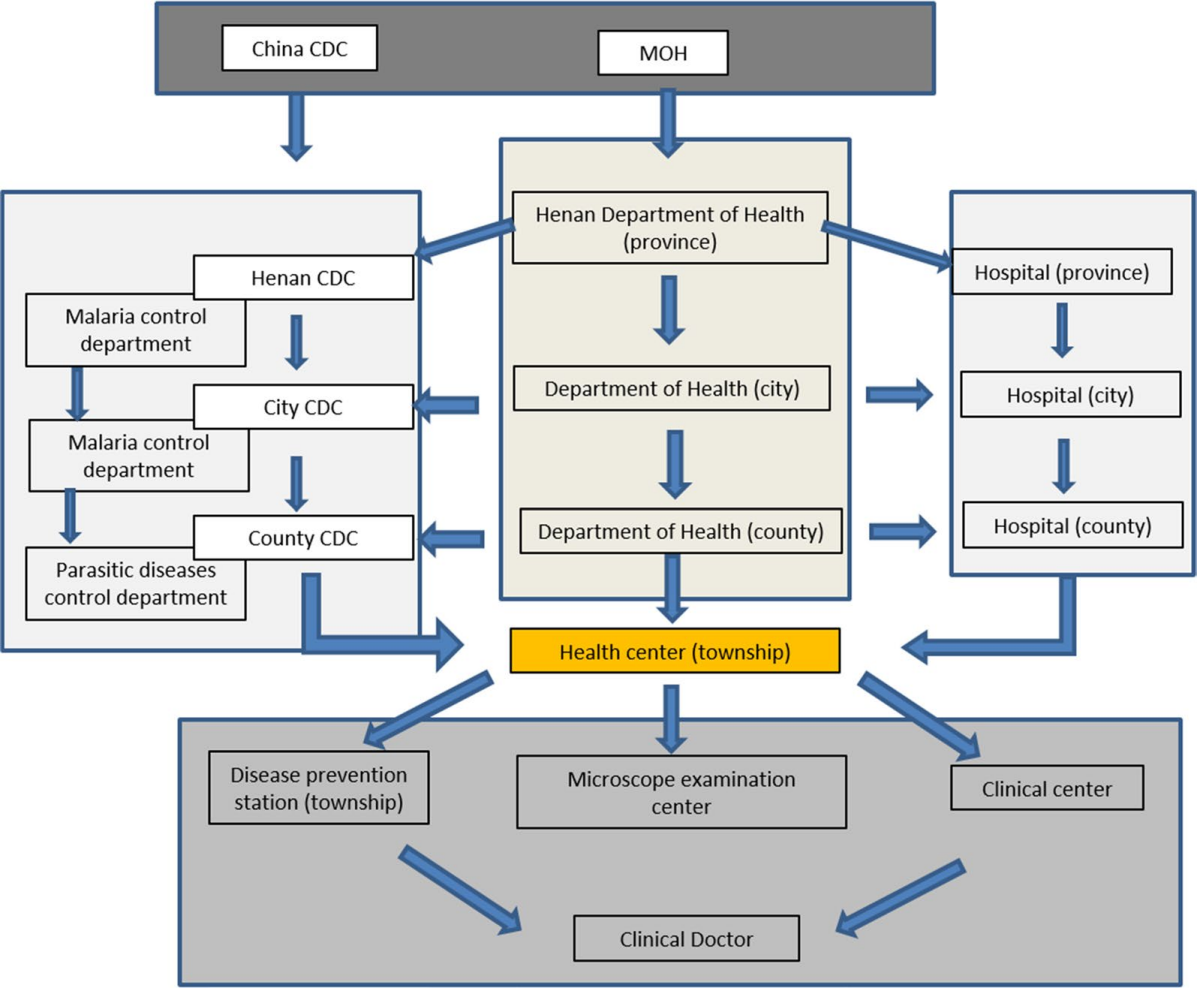
Malaria vertical prevention and control system with policy implementation and guidance: the malaria prevention and control network consists of professional prevention and control centers, government departments, and medical and health institutions at all levels. Under the direct leadership of the government departments at the same level, they undertake corresponding tasks

Malaria was once a serious public health problem in the Peopleʼs Republic of China (P. R. China) [1] with Henan province, located in the centre of the country, characteristic of endemic zones. Henan is in the transition zone from subtropical to warm temperate, and the history of malaria there can be traced back to 4000 years ago [1, 2]. Malaria used to be widespread in Henan with an unstable pattern of transmission, and with *Plasmodium vivax* accounting for more than 90% of malaria cases [3, 4]. Following the founding of the P. R. China, a notification system for infectious diseases was established. A total of 39 infectious diseases are notifiable by law and categorized as A, B or C diseases with malaria listed as a class B infection from 1956 onwards [5, 6]. Malaria control became part of a systematically organized disease prevention and control system with policy implementation and guidance organized vertically (Fig. 1). After the implementation of integrated malaria control and elimination programmes, the disease burden sharply declined, with no indigenous cases announced in 2017 at a national level [7].

**Fig. 2:**
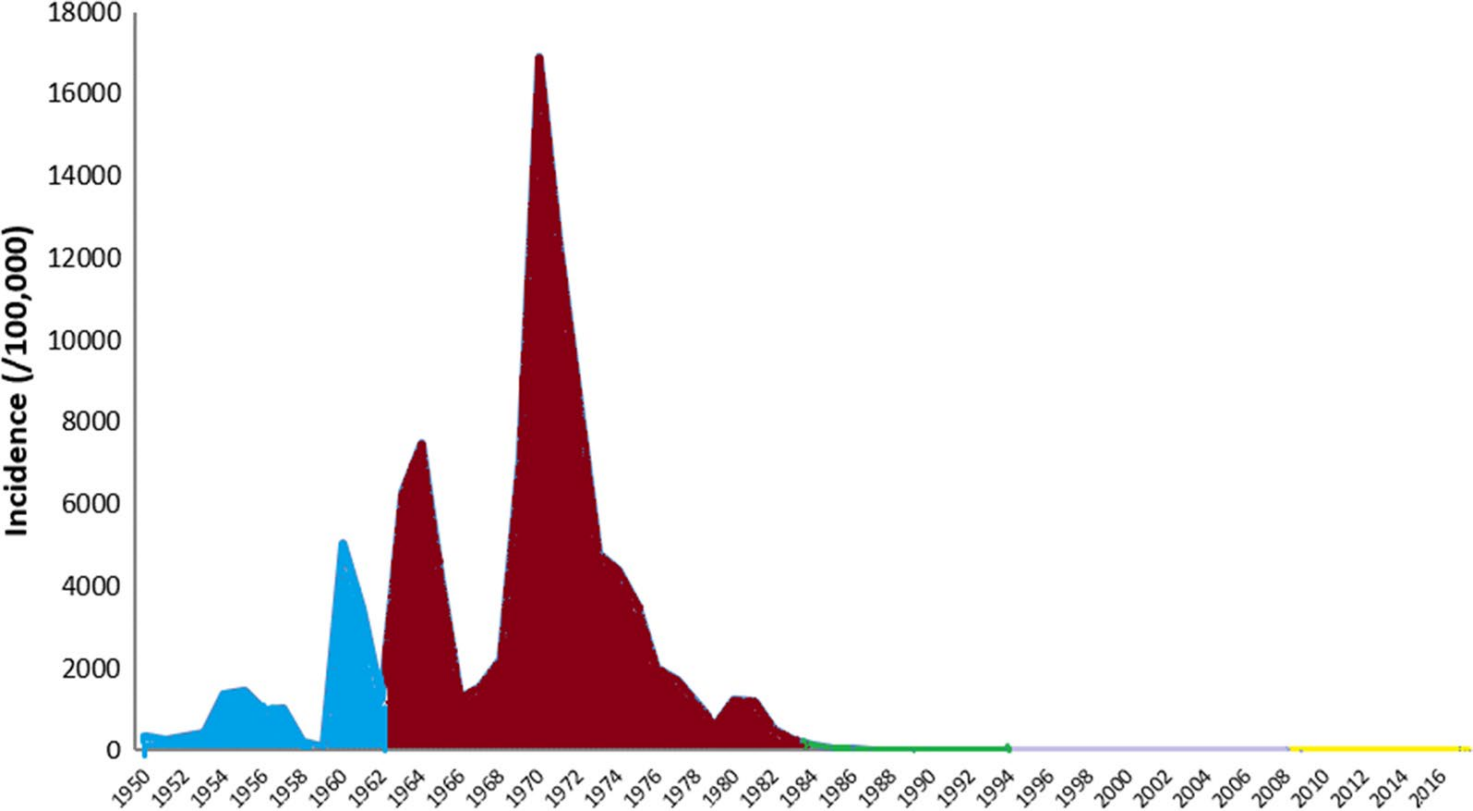
Malaria control history in Henan province between 1950 to 2017: (i) investigation and target intervention (1950–1962, blue); (ii) disease control (1963–1984, red); (iii) pre-elimination (1985–1994, green); (iv) consolidation (1995–2009, purple); and (v) elimination (2010–2020, yellow)

At the national level, the post-1949 history of malaria in China can be primarily grouped into five phases: (i) transmission unknown (1949–1959); (ii) outbreak and pandemic transmission (1960–1979); (iii) decline with sporadic cases (1980–1999); (iv) low transmission with re-emergence in central China (2000–2009); and, (v) the elimination phase (2010–2020) [7, 8]. In parallel with these phases at national level, malaria control in Henan experienced five stages: (i) investigation and target intervention (1950–1962); (ii) disease control (1963–1984); (iii) pre-elimination (1985–1994); (iv) consolidation (1995–2009); and (v) elimination (2010–2020) [1, 3, 4]. (Fig. 2).

The former Ministry of Health, along with 13 additional ministries, issued the National Malaria Elimination Action Plan (2010–2020) (NMEAP), with the objective of eliminating indigenous malaria in non-border regions before the end of 2015 and eliminating the disease nationwide before the end of 2020 [7]. Through decades of continuous malaria control activities, no indigenous malaria case has been reported since 2012 in Henan Province, 3 years ahead of the NMEAP target [9].

To understand the dynamics of this successful programme, data from one area in Henan that was monitored closely throughout this progression from control to elimination have been extracted. This paper summarizes the experiences and lessons of the programme, which could provide insights to other regions or countries with similar ecological settings aiming to move from malaria control to elimination.

## Methods

Historical data from a pilot study conducted in Guantang, Luyi in central China from 1971–1995, were extracted and digitized. All data is going to be summarized into four parts: study site, control and measurements, malaria data (including monthly numbers of reported cases, febrile cases, parasite carriage rates, the neonatal infection rate), and entomological data regarding *Anopheles sinensis*.

### Study site

**Fig. 3:**
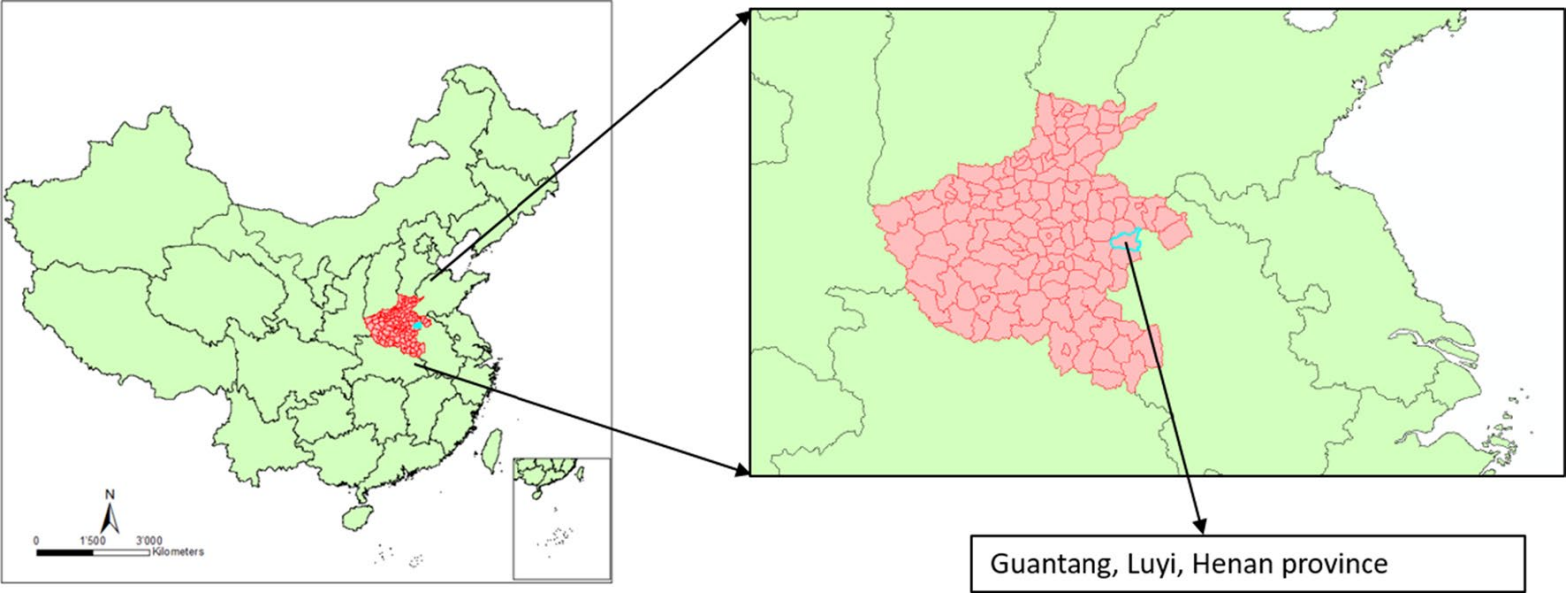
Study site of Guantang, Luyi, Henan province, P. R. China: red polygons are Henan province located in central China. Blue highlighted polygon is Luyi County. Southern part of Luyi is Guantang township

Guantang Township is located 10 km south of Luyi County, 33°51ʹ north latitude and 115°21ʹ east longitude, within the river network region of Henan province, bordering Anhui Province in the east (Fig. 3). There were 114 villages in the township with a population of about 40,000. In the early 1970s, about 4% of the cultivated land was planted with rice. Subsequently most arable land was replaced with dry land crops, such as cotton. Most of the houses were poorly ventilated bungalows with wood and mud structures. In summer and early autumn, residents generally had the habit of spending the evening outdoors or sleeping outside. These villages are crossed by more than 10 small but permanent rivers with considerable aquatic vegetation. In addition, there are over 200, mostly perennial, water bodies, of varying sizes, scattered within villages. The annual average temperature was 13.9–15 °C, and the annual average precipitation was 731. 8 mm. More than 63% of the precipitation is concentrated in June–September.

In 1970, a malaria outbreak in central China (including Henan province) drew the attention of multiple levels of the Chinese administration to the problem of malaria (Fig. 1) [8]. A pilot study in Guantang was established in 1971 to study patterns of *P. vivax* transmission, and served as a demonstration project for large-scale comprehensive malaria control in central China. Investigations and research on the epidemiology and control of malaria were carried out until 1995 [4].

### Main control measures

From 1971 to 1995, the pilot site continually received special funds and implementation guidance from a professional malaria control team. Integrated control methods were implemented throughout the province. The standard control strategy was: (1) immediate treatment of diagnosed patients with 3 days chloroquine (day 1: 600 mg, day 2: 300 mg and day 3: 300 mg) together with 5 or 8 days primaquine (150 mg for 5 days or 180 mg for 8 days) regimens or a 3-day-double-course therapy (chloroquine and primaquine base in divided doses); (2) in years with high incidence rates during the transmission season, mass prophylaxis with pyrimethamine was applied at the village level, while anti-malarial medication was applied in outbreak foci; (3) during the non-transmission seasons, patients with malaria infection history in the previous transmission season were treated to clear hypnozoites with different regimes at different time periods. Before mid-1970s, a regime of 8 days of primaquine (22.5 mg/dose) and 2 days pyrimethamine (50 mg/dose) was applied. After the mid-1970s, a regime of 3 days of chloroquine (day1: 600 mg, day 2: 300 mg and day 3: 300 mg) together with 5 or 8 days of primaquine (150 mg for 5 days or 180 mg for 8 days) was used. In the years with high incidence rates, a mass hypnozoite clearance programme was implemented at village level; (4) Mass anti-mosquito campaigns were carried out annually. There was no unified anti-mosquito technique applied in the province [4]. Only community-based anti-mosquito campaigns were carried out, which was one of the country's biggest "four pests"—rats, flies, mosquitoes, and sparrows campaigns introduced by Mao Zedong aiming to eradicate the transmission of pestilence and diseases [10]. In a few places with appropriate budgets, such as Guantang, larvicides, e.g. hexachlorocyclohexane powder, were applied to water bodies to eliminate mosquito larvae [4, 11].

### Malaria data collection

#### Malaria case reports

Confirmed malaria cases were reported at different levels of the surveillance system and hospitals. Due to institutional reforms, there are differing levels of available data over the period 1971–1995. Annual malaria case data of Guantang were available for each year from 1971 to 1995, while monthly data were only available from 1971–1984.

#### Febrile patients

Between 1979 and 1981, febrile patients with four classified groups, namely those presumptively diagnosed as malaria by symptoms, suspected malaria, unexplained fever or suspected cold, were verified by blood smears test, the gold standard for malaria cases [12]. Due to the typical fever pattern of *P. vivax* patient, a fever that recurs every second day [12], the fever pattern was used as one criteria for vivax malaria clinical diagnosis. The blood test results of four types of patients were counted and summarized.

#### Parasite rates of residents

Each year from 1971 to 1982 (except the year 1981) in the months of June and November, clustered sampling, such as school-, community-based sampling, accounting for 1 to 5% of whole township population, were carried out by blood smear test (finger-prick test) to check for malaria infection (Table 1). After 1983, this sampling investigation was discontinued due to low parasite rates and difficulty in collecting large amounts of blood samples.

**Table 1:** Parasitological surveys by clustered sampling in Guantang in the months of June and November from 1971 to 1982 (except the year 1981)

	June		November	
Year	No. positive cases	No. sampled people	No. positive cases	No. sampled people
1971	38	2018	10	813
1972	5	1141	8	1053
1973	3	1141	7	1139
1974	3	1113	2	2062
1975	1	458	1	293
1976	0	667	0	699
1977	0	441	3	429
1978	0	443	0	464
1979	0	431	0	300
1980	1	300	1	300
1982	0	503	0	542

#### Antibody survey

From 1978 to 1995 (with a few years omitted), the indirect fluorescent antibody (IFA) test [13] was used to examine malaria antibody levels within randomly selected residents.

#### Malaria infection in neonates

**Fig. 4:**
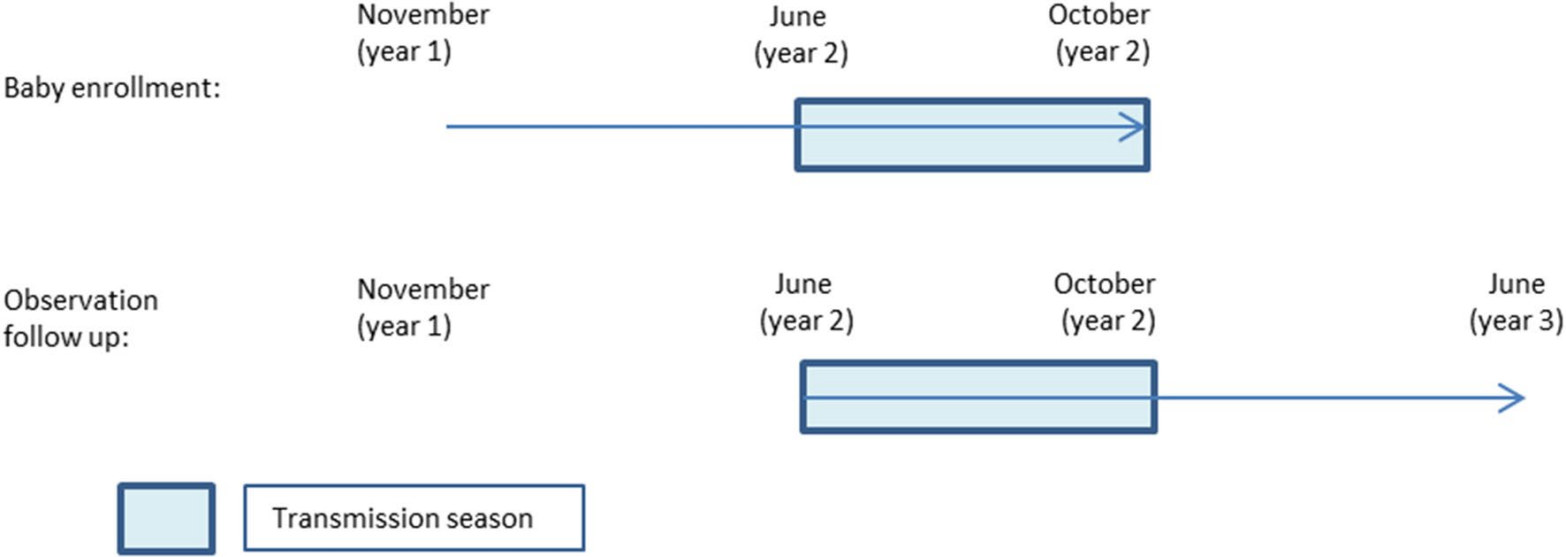
Schematic diagram of enrolment and following up of malaria infection in neonates: Malaria transmission season in Guantang is between June and October. Each enrolment started in November, the beginning of non-transmission season

From 1976 to 1981, infants born between November in previous year and October of the current year were required to undergo blood tests to identify any malaria infection if fever occurred during between beginning of the transmission season in June of the current year through to June next year (see Fig. 4). Malaria infection rates for the year were calculated given the confirmed cases in this newborn infants’ cohort. Those who experienced symptoms during the transmission season from June to December were recorded as short-latency episodes, while those who experience symptoms from January the following year until the next transmission season were classified as long-latency episodes.

### Entomological study of *Anopheles sinensis*

Mosquitoes were trapped using a variety of methods, differing in trap location (indoor/outdoor), the host used as bait (human and/or cattle), time and duration [e.g. 2 h after sunset/overnight (8 pm–4 am)]. *Anopheles sinensis* was the only malaria vector found in the pilot study area [11]. No other *Anopheles* mosquito species were detected.

#### Human and cattle outdoor trapping for 2 h after sunset

For each year between 1971 and 1984, every 10 days during the transmission season from the beginning of June until the end of October, fixed-point outdoor mosquito landing collections were carried out in parallel on both human and cattle. The two mosquito traps were separated by 100 m and mosquitoes were caught continuously for 2 h after sunset.

#### Human indoor/outdoor overnight trapping

Once a month in the years 1976, 1977 and 1982, from June to September, indoor human mosquito landing collections were carried out from 8 pm until 4 am and the number of mosquitoes was recorded. In addition, fixed-point outdoor human landing collections were conducted from 8 pm until 4am every 10 days from June until the end of October in 1975 through to 1984.

#### Habitats of indoor resting mosquitoes

*Anopheles sinensis* uses a wide range of resting places. Between 1971 and 1975, from May to October each year, female mosquitoes were caught by a person for 15 min inside various buildings, including bedrooms, cattle stalls, pig stalls, garages, other rooms, and brick kilns.

#### Blood-feeding behaviour

In 1975 and 1978, newly blood-fed female mosquitoes in different types of habitats in the wild were caught. Blood samples were tested by an electrophoresis antigen–antibody reaction to define blood-feeding hosts [14]. From June to September of 1975, female mosquitoes under the bridge Zhouqiao near Guantang village were captured to check blood-fed specimens. The stage of blood meal digestion within a mosquito was classified according to the Sella scale [4].

#### Vectorial capacity

Vectorial capacity is often used to express malaria transmission risk by local vector populations (see formular below). Between 1975–1984, vectorial capacity (C) from June to September was estimated based on the Garrett-Jones’s original Eq. (1) [15] by local malaria health workers:

1 $$C\, = \,\frac{{ma^{2} p^{n} }}{ - \ln (p)}$$

where *m* is the ratio of mosquitoes to humans; *a* the rate at which mosquitoes bite humans (per day) biting rates; *n* the parasite's extrinsic incubation period (EIP, *n* days); and *p* is the mosquito survival through one day.

## Results

**Fig. 5:**
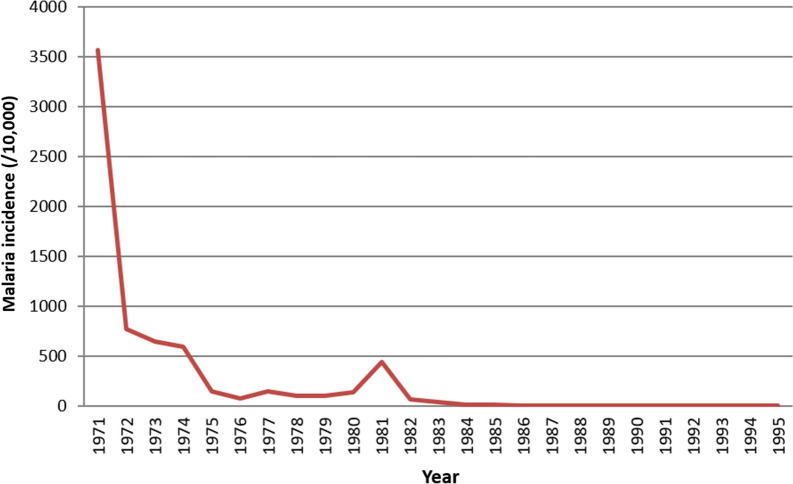
Malaria incidence rate in Guantang 1971–1995. A fluctuation observed in 1981

Following 25 years of significant effort, the incidence of malaria in Guantang decreased from 4,333 cases per 10,000 people in 1970 before integrated implementation to 0.23/10,000 in 1991, and no cases observed between 1992–1995. There were some epidemic fluctuations from 1977 to 1981 (Fig. 5). During the study period the estimated parasite rate of residents, measured antibody levels and the estimated neonatal infection rate also decreased, indicating that the integrated malaria control measures effectively controlled malaria and achieved the goal of malaria elimination.

### Febrile patients

From 1979 to 1981, parasite positive rates of febrile patients, with diagnosis of malaria by symptom, suspected malaria, unexplained fever or suspected cold, were 53.2% (666/1252), 47.4% (704/1485), and 9.1% (117/ 1286) and 13.3% (242/1822), respectively (See Table 2). Among all 1,729 malaria cases, malaria symptom-based diagnosis correctly detected only 666 cases (38.5%). Taking into consideration microscopy as malaria diagnosis gold-standard, 61.5% (1063/1729) malaria infections were underreported and 46.8% (586/1252) non-malaria cases were falsely reported as malaria infections. Prior to this period, reports of malaria cases in other places of Henan province were mainly based on clinically symptomatic diagnosis.

**Table 2:** *Plasmodium vivax* positive rates of febrile patients with diagnosis of malaria by symptom, suspected malaria, unexplained fever or suspected cold in Guantang between 1979 and 1981

	Febrile patient groups				
	Malaria clinical symptom	Suspected malaria	Unexplained fever	Suspected cold	Total
No. cases	1252	1485	1286	1822	5845
No. confirmed by microscope	666	704	117	242	1729
Parasite positive rates	53.2%	47.4%	9.1%	13.3%	

### Recurrent malaria

Malaria cases during the non-transmission seasons from 1972 to 1987 were interviewed and recorded. Most malaria cases (78.3%) in the non-transmission season exhibited no symptoms in the previous transmission season (Table 3).

**Table 3:** Malaria cases investigation in non-transmission season in Guantang between 1971 and 1987

Year	No. cases in non-transmission season	Without malaria symptom in previous transmission season	Ratio (%)
1972	540	227	42.04
1973	663	510	76.92
1974	705	676	95.89
1975	160	127	79.38
1976	163	151	92.64
1977	45	43	95.56
1978	112	102	91.07
1979	100	85	85.00
1980	134	111	82.84
1981	241	198	82.16
1982	64	50	78.13
1983	49	44	89.80
1984	19	19	100.00
1985	3	3	100.00
1986	0	0	–
1987	1	1	100.00
Total	2999	2347	78.3

Each year from 1971 to 1984, a certain number of malaria cases in the transmission season were followed up to observe recurrent rates. Among 14,320 malaria cases followed, less than 6% had recurrent malaria during the following non-transmission season (Table 4).

**Table 4:** Recurrent rate of malaria cases in transmission season in Guantang between 1971 and 1984

Year	No. malaria in transmission season	No. recurrent cases in non-transmission season	Ratio (%)
1971	5804	90	1.55
1972	2200	313	14.23
1973	1654	153	9.25
1974	1498	29	1.94
1975	400	33	8.25
1976	89	12	13.48
1977	506	2	0.40
1978	270	10	3.70
1979	297	15	5.05
1980	396	23	5.81
1981	899	43	4.78
1982	177	14	7.91
1983	107	5	4.67
1984	23	0	0.00
Total	14,320	742	5.18

### Parasite carriage

The carriage rate of asexual blood-stage parasite in residents in the beginning and end of transmission season were very similar. Although parasite carriage fluctuated between years, overall it declined from 1.7% in 1971 to 0 in the 1980s. Of the 7,634 clinically symptomatic patients who were positively confirmed by blood smear tests between 1977 and 1982, gametocytes were detected in 5,251 (68.8%). In addition, among the 59 confirmed cases with first clinical episodes, gametocytes were detected in 55 on the first day of symptoms. The carriage rate of gametocytes was high during this period.

### Antibody survey

According to the documented reports, the antibody positivity was higher before 1984. Since 1985, about 1% of the population were positive for malaria antibodies by IFA (Table 5).

**Table 5:** Malaria antibody levels within randomly selected residents in Guantang between 1978 and 1995 the indirect fluorescent antibody (IFA) test (except years 1981–1983 and 1987)

Year	No. people tested	No. people positive	Positive rate (%)
1978	907	80	8.82
1979	611	51	8.35
1980	524	72	13.74
1984	269	11	4.09
1985	300	1	0.33
1986	627	2	0.32
1988	400	0	0
1989	282	0	0
1990	300	0	0
1991	400	5	1.25
1992	331	3	0.91
1993	845	8	0.95
1994	862	6	0.70
1995	393	6	1.53

### Malaria infection in neonates

From 1976 to 1981, among 4,858 babies examined for malaria infection, 41 malaria cases were confirmed by blood smear test of which 25 cases occurred between July to December of the year enrolled, and 13 cases in the non-transmission period of the following year (See Table 6). The ratio of long-term to short-term incubation is therefore 1:2.15.

**Table 6:** Incubation period study of primary infection among neonates malaria in Guantang between 1976 and 1981

Year	No. total enrolled neonates	No. total cases	Month											
			7	8	9	10	11	12	1	2	3	4	5	6
1976	750	1					1							
1977	917	6		2	1								1	2
1978	1014	5			1	4								
1979	909	6		3	2						1			
1980	664	16		1	1	3	2				3		5	1
1981	604	7	3	2	2									
Total	4858	41	3	8	7	7	3				4		6	3

### Entomological characteristics of Anopheles sinensis

#### Seasonal density

**Fig. 6:**
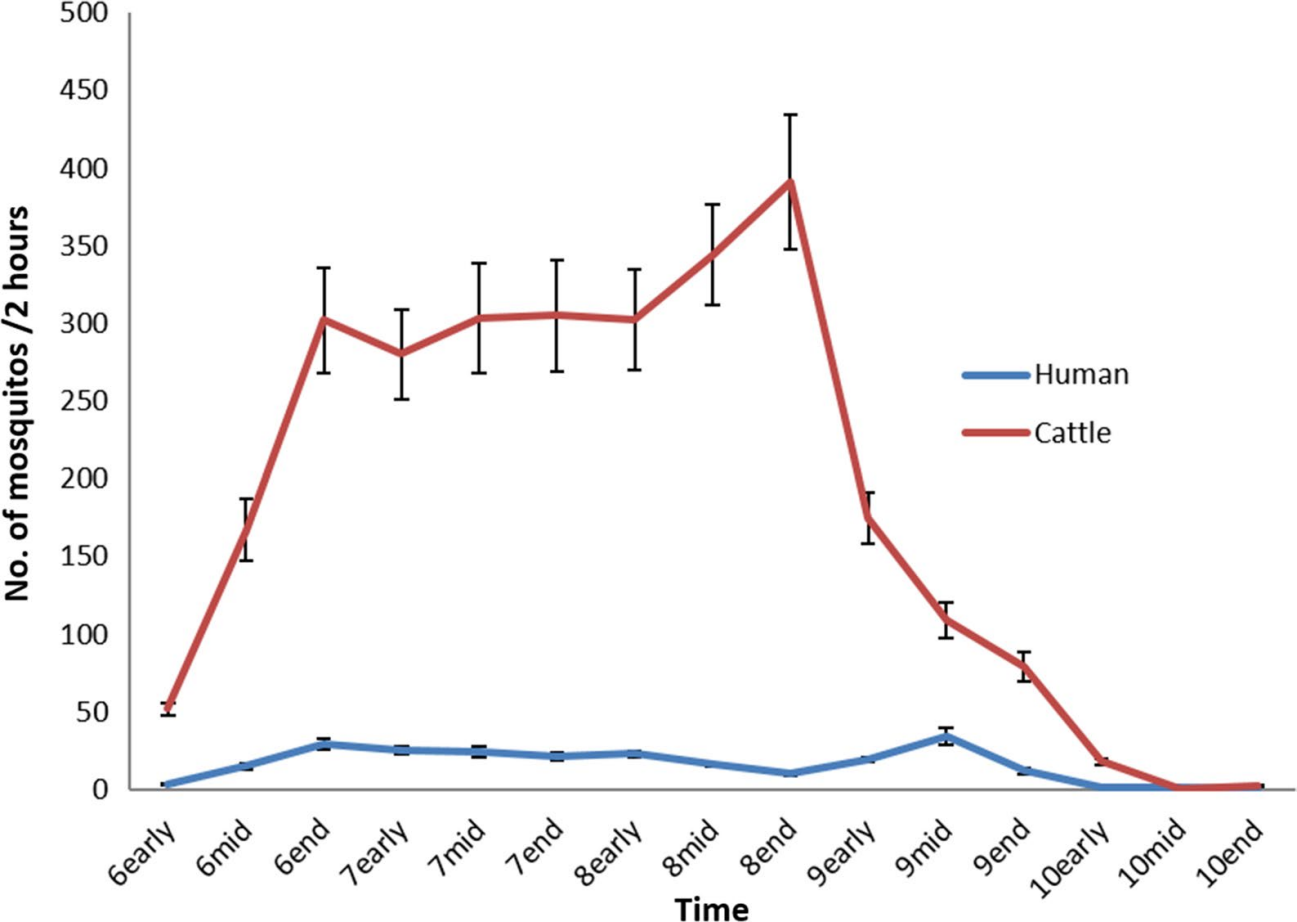
Fixed-point outdoor mosquito landing collections on both human and cattle by every 10 days during the transmission season from the beginning of June until the end of October: solid line is the averaged number of mosquitos between 1971 and 1984 with 95% confidence interval

The number of mosquitoes trapped by either outdoor human and cattle traps between years 1971 and 1984 indicated that the density of mosquitoes was likely very low in late May or early June, with the first density peak in late June, and a second one in late August. The density dropped sharply in September and was too low to measure precisely in October (Fig. 6). Other observations also showed that development of the ovaries of female mosquitoes ceased in late November [4, 11].

### Blood-feeding behaviour

*Host preferences:* When searching for blood hosts, *An. sinensis* expresses a preference for cattle, with an estimated average ratio of 1:11.9 between human and cattle in paired landing catches (Fig. 6). Most of the indoor resting mosquitoes were captured in cattle and pig stalls.

*Indoor/outdoor preference:* The ratio of number of mosquitoes captured by human indoor / outdoor overnight landing collections was 1:7.73, indicating that *An. sinensis* prefers outdoor blood-feeding (Table 7).

**Table 7:** Number of mosquitos captured by human indoor/outdoor overnight landing collections in different months

Year	June		July		August		September	
	Indoor	Outdoor	Indoor	Outdoor	Indoor	Outdoor	Indoor	Outdoor
1976	3	13	6	44	3	11	0	1
1977	1	9	3	60	4	16	1	10
1982	0	1	0	0	1	2	0	4

*Blood-feeding activity overnight:* Blood-feeding activities of *An. sinensis* persist throughout the night. Although there were some variations between different months, generally two blood-feeding peaks can be detected. One was around 8 pm, 2 h after sunset. The other one happens between 11 pm and 2am (Table 8).

**Table 8:** Mosquito blood-feeding activity over night from 8 pm to 3 am by indoor human mosquito landing collections from June to September: the number of mosquitos presented in each cell is the averaged value between 1975 and 1984

	No. mosquitos/person.night			
Time	June	July	Aug	Sept
8:00 pm	11	20	13	17
9:00 pm	11	13	11	11
10:00 pm	3	18	11	10
11:00 pm	13	20	12	7
12:00 pm	26	22	14	3
1:00 pm	15	24	20	6
2:00 pm	8	26	12	10
3:00 pm	6	13	7	4
4:00 pm	3	14	4	5

There is no original data available for each year

*Human blood index:* In 1975 and 1978, 170 and 433 newly blood-fed female mosquitoes in different types of habitats in the wild were caught. Of these, 44 and 132 were human blood positive in 1975 and 1978, respectively, corresponding to an average human blood index of 0.29.

*After blood-feeding:* Among the 523 *An. sinensis* captured under the bridge in Zhouqiao, the gastric blood of Sella scale II-III, accounted for 30.8%, and IV-V scale accounted for 38.8%.

*Sporozoite rate:* From June to August in 1974, a total of 2,068 *An. sinensis* were dissected. Only one mosquito’s salivary gland was positive for sporozoites corresponding to a natural infection rate of 0.05%.

**Fig. 7:**
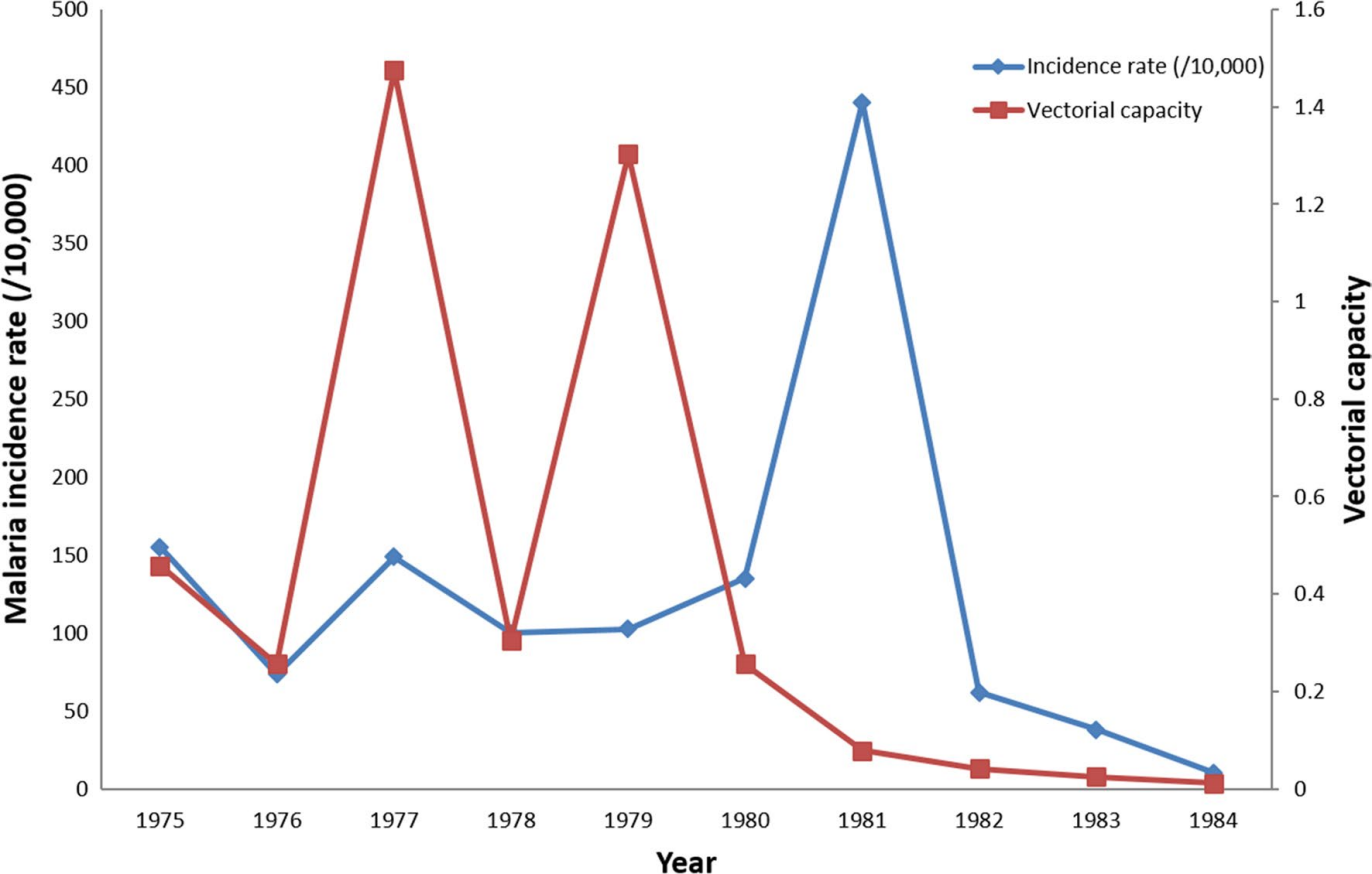
Malaria incidence rate vs vectorial capacity in Guantang between 1975 and 1984: vectorial capacity is provided by historical data

*Vectorial capacity:* Between 1975 to 1984, a substantial fluctuation in vectorial capacity was observed with peaks in 1977 and 1979. After 1980, vectorial capacity showed a continuously declining trend (Fig. 7).

## Discussion

*Plasmodium vivax* was the major malaria species in Henan province and responsible for more than 90% of the local malaria [3]. Transmission was unstable with several outbreaks in 1950s, 1960s and 1970s. From the 1980s onwards, although the incidence rate was low, there was considerable inter-annual variation especially between 1985 and 2009 [16]. Henan Province, and Guantang in particular, is located in the warm temperate zone with a clear seasonal pattern of transmission, with the transmission season usually spanning June to October. From November onwards, low ambient temperatures inhibited mosquito movement and survival, preventing transmission. However, due to global warming, the temporal limits of mosquito survival have expanded, prolonging the potential malaria transmission season [17]. The central part China (i.e., Anhui, Henan and Jiangsu provinces) form a climate sensitive zone [17] that experienced re-emergence of malaria between 2000 and 2006 and the surveillance response system needs to continue to be consolidated in this specific region [1, 18].

The malaria parasite phenotype in Guantang was found to be similar to that of *P. vivax* described as North Korean [19], but the Guantang analysis indicates the primary episode and latency of over 12 months is relatively longer than *P. vivax* North Korean strains, with relapses occuring roughly 8–9 months after primary infection [19]. Experiments conducted by Le-Yuan Shang found that incubation periods of *P. vivax* in Henan Province could be up to 443 days long (~ 15 months) (238–443 days) [4]. Among the malaria cases in Henan from January to May (non-transmission season) in 1972–1987, those without malaria symptoms in the previous year accounted for more than 76.9% (2,347/2,999) which indicates that a long incubation period (latency) malaria was dominant in the non-transmission period.

A study carried out by Coatney suggests that the main factor determining duration of latency or the inter relapse interval (for the first relapses with genetically homologous parasites) was inoculum size of sporozoites rather than immunity [20]. A further study conducted by Le-Yuan Shang confirmed that Henan Province had predominately long incubation type *P. vivax* (temperate) with a ratio of long- to short-term incubation infections in neonates of 1:2.15, similar to the outcomes of infections with a high inoculum size in adults [4].

Owing to the long latency in Henan, clearing hypnozoites in asymptomatic hosts by mass treatment with primaquine was very likely a driver and thus of considerable importance in reducing the reservoir of infection, which would otherwise have been the main contributor in the following transmission season. This finding provided a scientific basis for the implementation of mass campaigns of liver stage hypnozoite clearance in Henan province especially in the years that experienced malaria outbreaks, such as 1960s and 1970s. However, in low-endemic areas this strategy may not be cost-effective, since mass treatment in non-transmission seasons to support hypnozoite elimination entails targeting a large number of people, a large demand for drugs, and enormous resources in terms of manpower, all of which requires in-depth organization.

There is the additional risk of clustered adverse reactions [21], and the approach is ethically controversial if only a small number of the population are carrying hypnozoites or at risk. Therefore, He et al*.* suggested an alternative strategy to mass treatment that a) in a village with incidence rate of  > 3%, positive episodes and the residents within 50 m radius take a 3-day- chloroquine and a 5 or 8-day-primaquine regimen treatment; b) in a village with incidence rate of  < 3%, only positive episodes receive a 3-day-chloroquine and a 5 or 8-day-primaquine regimens; c) during non-transmission seasons a 4-day-chloroquine and primaquine treatment could be given, but only targeted on people with malaria infection history [21]. When scholars in China found that 86% of malaria cases are distributed within 100 m of the main water body [22], it was proposed that the scope of the hypnozoite clearance programme should be defined by location of detection, the distance to water body and the living conditions of neighbourhood residents. This strategy was assessed in a pilot study in Anhui Province with significant success [22].

Episodes of recurrent parasitaemia following treatment may be due to recrudescence of the initial infection, reflecting failure of the drug to clear the infection, or may be due to re-infection, or due to relapse by activated hypnozoites. Since the causes in Henan were not distinguishable with available data, only episodes in the non-transmission season could be distinguished. For most years, malaria episodes in the non-transmission season with documented history of malaria accounted for less than 10% of total episodes in the previous transmission season, with an average recurrent rate of 5.18% (Table 4). Thus a 3-day-chloroquine (1200 mg) and 5 or 8-day-primaquine (150 or 180 mg) administration efficiently control the recurrence rate of malaria in the pilot area. Assuming complete clearance of parasites in the blood by chloroquine, 5.18% cases were still attributed to drug resistance to primaquine. This is slightly higher than the report of 4.5% by Saifi with administration with 1500 mg chloroquine and 75 mg primaquine [2].

The average HBI of outdoor mosquitoes in Guantang in 1970s was 0.29, indicating that *An. sinensis* is an animal preferring species, similar to findings from a study carried out in Nanjing, Jiangsu Province in the period of 1952–1955 with HBI 0.26 by testing 2616 *An. sinensis* [14]. Field investigations in northern Anhui in August and September, 2005 showed that HBI of *An. sinensis* was 0.18 [18, 23]. Studies carried out in 4 counties in Yunnan Province between June and August, 2012 detected low HBI of 0.018 [24]. There is thus large variation in the estimates of HBI obtained by these different surveys. However, a clear declining trend in time of the estimated HBI corresponds to a period of economic growth and improved rural living conditions such as mosquito meshes installed in doors and windows and popularity of electric fans and air conditioning. The habit of sleeping outdoors in summer has also changed. In Guantang, the *An. sinensis* favoured habitats such as rice fields have been gradually modified for cultivation to dry land products, such as cotton. This agricultural change has undoubtably significantly reduced contact between mosquitoes and people. The Sella scale is the indicator of mosquito gastric blood digestion degree with a lower Sella scale indicating shorter time after blood-feeding. In Guantang, around 1/3 of mosquitoes were at the Sella scale II-III, which means *An. sinensis* belongs to the outdoor-resting mosquito species. After blood-feeding on various hosts, mosquitoes were flying to the outside of the village for oviposition.

### New challenges

In areas of China with unstable transmission, epidemics on a 10 year periodicity were observed in the early 1950s, 1960s, 1970s, and 1980s [1]. These cycles have been attributed to either a regular pattern of solar activity cycle which may have an impact on regional climate and weather; or to intrinsic cycles in the dynamics of *P. vivax* malaria resulting from activation of long dormancy hypnozoites [21]. The latter hypothesis is more consistent with the finding that the outbreak in 1981 was not associated with any increase in vectorial capacity (Fig. 7). Challenges remain in the maintenance of non-transmission status owing to the long dormancy of liver stage hypnozoites.

If ecological conditions remain unchanged and *Anopheles* mosquitoes remain widespread, once intervention activities cease there is always potential for malaria to be re-introduced and resurge. As recently as 2015, officials reported two local cases of *P. vivax* [25] in Liaoning, where malaria transmission had been interrupted for 4 years. In recent years, the number of recorded imported falciparum malaria cases has increased with most patients being residents returning from business, tourism or work in malaria highly endemic regions, i.e. Africa and Southeast Asia [7, 26]. To mitigate risks of reintroduction, the Henan Provincial Health Department in conjunction with other relevant public bodies, continue to conduct malaria surveillance and response activities involving tracking malaria cases, screening key populations and communicating related information to control imported *P. falciparum* malaria [16].

### Conclusion

The integrated malaria control approach in Guantang effectively controlled malaria and achieved elimination. Analysis of the effectiveness of the programme can provide guidance to other regions or countries with similar ecological settings aiming to move from malaria control to elimination. There is a potential challenge in the maintenance of non-transmission status owing to imported cases and the long dormancy of liver stage hypnozoites.
